# Glucose deficit triggers tau pathology and synaptic dysfunction in a tauopathy mouse model

**DOI:** 10.1038/tp.2016.296

**Published:** 2017-01-31

**Authors:** E Lauretti, J-G Li, A Di Meco, D Praticò

**Affiliations:** 1 Department of Pharmacology and Center for Translational Medicine, Lewis Katz School of Medicine, Temple University, Philadelphia, PA, USA

## Abstract

Clinical investigations have highlighted a biological link between reduced brain glucose metabolism and Alzheimer's disease (AD). Previous studies showed that glucose deprivation may influence amyloid beta formation *in vivo* but no data are available on the effect that this condition might have on tau protein metabolism. In the current paper, we investigated the effect of glucose deficit on tau phosphorylation, memory and learning, and synaptic function in a transgenic mouse model of tauopathy, the h-tau mice. Compared with controls, h-tau mice with brain glucose deficit showed significant memory impairments, reduction of synaptic long-term potentiation, increased tau phosphorylation, which was mediated by the activation of P38 MAPK Kinase pathway. We believe our studies demonstrate for the first time that reduced glucose availability in the central nervous system directly triggers behavioral deficits by promoting the development of tau neuropathology and synaptic dysfunction. Since restoring brain glucose levels and metabolism could afford the opportunity to positively influence the entire AD phenotype, this approach should be considered as a novel and viable therapy for preventing and/or halting the disease progression.

## Introduction

Alzheimer's disease (AD) and related tauopathies are neurodegenerative disorders pathologically defined by the presence of abundant and highly phosphorylated forms of the microtubule-associated tau protein which later aggregates into fibrils and finally forms the neurofibrillary tangles (NFTs).^1^ Although it is known that the presence and abundance of NFTs correlates with the severity of dementia and neuronal loss,^2, 3^ the mechanisms leading to the abnormal high phosphorylation of tau in the brain of these patients remain unclear.

Consistent evidence has provided support for the notion that that exposure to physiological and psychological stressors can trigger tau phosphorylation in rodents. Among the different type of stress, in recent years a lot of attention has been devoted to the relationship between metabolic stress and brain function.^4, 5^ Despite the fact that the brain can use ketone bodies in order to maintain its basal functions, glucose is the main source of energy for the organ and its depletion has been shown to induce endoplasmic reticulum (ER) stress.^5^

Glucose deprivation can occur in a variety of conditions including cerebral ischemia, aging and neurodegenerative diseases.^6, 7^ Interestingly, positron emission tomography imaging studies have shown that glucose utilization is lower in AD than in age-matched healthy control brains.^8^ In support to this observation, previous studies have demonstrated that in the transgenic mice Tg2576 (overexpressing the Swedish mutant of human APP), energy metabolism inhibition causes a post-transcriptional increase in BACE-1 levels, which leads to elevated Aβ formation and deposition.^9^ On the other hand, we have previously reported that in response to glucose deprivation neuronal cells manifest an increase in tau phosphorylation via the activation of the P38 MAPK pathway.^10^ This *in vitro* observation supported the novel hypothesis that energy deprivation may also have a role in the development of tau neuropathology, the second most important hallmark lesion of the AD brain. However, to the best of our knowledge, so far no data are available supporting these *in vitro* findings and their functional significance by demonstrating that indeed a condition of glucose deprivation by increasing tau phosphorylation will result in memory deficit and synaptic dysfunction *in vivo*.

In the current study, we used a pharmacological model of glucose deprivation and investigated its effect on tau phosphorylation, synaptic function and cognition in a relevant transgenic mouse model of tauopathy, the h-tau mouse. Under our experimental condition, we found that, compared with controls, glucose-deprived mice had impaired memory and reduced synaptic long-term potentiation, which associated with a significant increase in tau phosphorylation and apoptosis.

Taken together, to our knowledge, our findings provide the first *in vivo* evidence that this metabolic stressor by influencing tau metabolism is a pleiotropic and active modulator of the pathogenesis of AD and related tauopathies.

## Materials and methods

### Animals and treatment

All animal procedures were approved by the Institutional Animal Care and Usage Committee, in accordance with the US National Institutes of Health guidelines. The h-tau mouse model implemented in this study was previously described.^11^ Briefly, the mouse line, designed to express only human tau, was generated by crossing of 8c tau mice, which express all human tau isoforms, and tau knock-out mice. Animals were kept in a pathogen-free environment, on a 12-h light/dark cycle and fed a normal chow and water *ad libitum*. Male and female mice were used throughout the studies and randomized into two groups: control (CTR) and 2-deoxyglucose (DG). Starting at 6 months of age, each mouse was administered with an intraperitoneal injection of 1 × PBS (CTR) (*n*=10), or 1 g kg^−1^ 2-Deoxyglucose (DG) (*n*=10) (Sigma, St. Louis, MO, USA) once a week. When they reached 10 months of age, mice underwent behavioral testing as described below and then euthanized. After perfusion brains were removed, gently rinsed in cold 0.9% phosphate-buffered saline and immediately dissected in two halves. One half was immediately stored at −80 °C for biochemistry; the other half was fixed in 4% paraformaldehyde in phosphate-buffered saline, pH7.4 for immunohistochemistry studies.

### Behavioral tests

All the animals were handled for at least 3–4 consecutive days before testing. They were tested in random order and the experimenter conducting the tests was unaware of the treatment.

### Y-maze

The Y-maze apparatus consisted of 3 arms 32 cm (long) 610 cm (wide) with 26 cm walls (San Diego Instruments, San Diego, CA, USA). Testing was always performed in the same room and at the same time of the day to ensure environmental consistency, as previously described.^12^ Briefly, each mouse was placed in the center of the Y-maze and allowed to explore freely through the maze during a 5-min session for the assessment of spontaneous alternating behavior. The sequence and total number of arms entered were video-recorded. Any entry into an arm was considered valid if all four paws entered the arm. An alternation was defined as three consecutive entries in three different arms (1,2,3 or 2,3,1 and so on). The percentage alternation score was calculated using the following formula: total alternation number/total number of entries−2) × 100.

### Morris water maze

A white circular plastic tank (122 cm in diameter) with walls 76 cm high, filled with water maintained at 22°±2 °C, made opaque by the addition of a nontoxic white paint, was used to perform the Morris water maze test, as previously described.^13^ Briefly, mice were trained to swim to a submerged Plexiglas platform from 4 different starting points, on a daily basis for a total of 5 days. If they failed to find the platform within 60 s, they were manually guided to the platform and allowed to remain there for 15 s. Mice were trained to reach the training criterion of 20 s (escape latency). Mice were assessed in the probe trial 24 h after the last training session. The probe consisted in a free 180 s swim in the pool without platform. Each animal's performance was monitored using the Any-Maze video tracking system, which provided data for the acquisition parameters (latency to find the platform and distance swam and) and the probe trial parameters (number of entries in the target platform zone of the platform and time in quadrants).

### Immunoblot analyses

Primary antibodies used in the paper are summarized on Table 1. Immunoblot analyses were performed as previously described.^10, 12^ Briefly, proteins were extracted in enzyme immunoassay buffer (RIPA) containing 250 mM Tris base, 750 mM NaCl, 5% NP-40, 25 mM EDTA, 2.5% sodium deoxycholate, 0.5% sodium dodecyl sulfate and an EDTA-free protease and phosphatase inhibitors cocktail tablet (Roche Applied Science, Indianapolis, IN, USA), sonicated, centrifuged at 45 000 r.p.m. for 45 min at 4 °C, and supernatants used for the analysis. Total protein concentration was determined by using BCA Protein Assay Kit (Pierce, Rockford, IL, USA). Samples were electrophoretically separated using 10% Bis–Tris gels or 3–8% Tris–acetate gel (Bio-Rad, Richmond, CA, USA), according to the molecular weight of the target molecule, and then transferred onto nitrocellulose membranes (Bio-Rad). They were blocked with Odyssey blocking buffer for 1 h; and then incubated with primary antibodies overnight at 4 °C. After three washing cycles with T-TBS, membranes were incubated with IRDye 800CW or IRDye 680CW-labeled secondary antibodies (LI-COR Bioscience, Lincoln, NE, USA) at 22 °C for 1 h. Signals were developed with Odyssey Infrared Imaging Systems (LI-COR Bioscience). Actin was always used as an internal loading control.

### Formic acid insolubility assay

Mouse brain homogenates were sequentially extracted first in RIPA for the tau soluble fractions and then in formic acid (FA) for the tau insoluble fractions as previously described.^12, 13^ Insoluble fractions were then immunoblotted with HT-7 antibody.

### Immunohistochemistry

Primary antibodies used in this study are listed on Table 1. Immunostaining was performed as reported previously.^12, 13^ Briefly, serial 6-μm thick coronal sections were mounted on 3-aminopropyl triethoxysilane-coated slides. Every eighth section from the habenular to the posterior commissure (8–10 sections per animal) was examined using unbiased stereological principles. The sections for testing total tau (HT-7), phospho-tau (PHF-1, AT8, AT270), synaptophysin (SYP) and microtubule-associated protein-2 (MAP-2) were deparaffinized, hydrated, subsequently pretreated with 3% H_2_O_2_ in methanol and then treated with citrate (10 mm) for antigen retrieval. Sections were blocked in 2% fetal bovine serum and then incubated with primary antibody overnight at 4 °C. The following day, sections were incubated with biotinylated anti-mouse immunoglobulin G (Vector Laboratories, Burlingame, CA, USA) and then developed by using the avidin–biotin complex method (Vector Laboratories) with 3,3′-diaminobenzidine as a chromogen. Consecutives sections were incubated in the absence of primary antibodies to ensure specificity of staining.

### Electrophysiology

Ten-month-old mice ((*n*=No. of slices/No. of animals): h-tau (*n*=6/4); h-tau+DG (*n*=6/4)) were euthanized by rapid decapitation and brains placed into ice-cold artificial cerebral spinal fluid (ACSF) in which sucrose (248 mm) was substituted for NaCl. Analysis was performed as previously described.^14^ Briefly, transverse hippocampal slices (400-μm thick) were cut using a Vibratome 3000 plus (Vibratome, Bannockburn, IL, USA) and placed in ACSF (124 nm NaCl, 2.5 mm KCl, 2 mm NaH_2_PO_4_, 2.5 mm CaCl_2_, 2 mm MgSO_4_, 10 mm dextrose and 26 mm NaHCO_3_) at room temperature to recover for 1 h and bubbled with 95% O_2_/5% CO_2_. Slices were transferred to a recording chamber (Warner Instruments, Hamden, CT, USA) and continuously perfused with ACSF at 1.5–2.0 ml min^−1^ flow, bubbled with 95%O_2_/5% CO_2_ and maintained by an in-line solution heater (TC-324; Warner Instruments) at 32–34 °C. Field excitatory postsynaptic potentials (fEPSPs) from the CA1 stratum radiatum were recorded by using an extracellular glass pipette (3–5 MΩ) filled with ACSF. Schaffer collateral/commissural fibers in the stratum radiatum were stimulated with a bipolar tungsten electrode placed 200–300 μm from the recording pipette. Stimulation intensities were chosen to produce a fEPSP that was 1/3 of the maximum amplitude, based on an input/output (I/O) curve using stimulations of 0–300 μA, in increments of 20 μAs. Paired-pulse facilitation experiments were performed using a pair of stimuli of the same intensity delivered 20, 50, 100, 200 and 1000 ms apart. Baseline was recorded for 20 min before tetanization with pulses every 30 s. Long-term potentiation (LTP) at CA3–CA1 synapses was induced by four trains of 100 Hz stimulation delivered in 20 s intervals. Recordings were made every 30 s for 2 h following tetanization. The fEPSP rise/slope (mV ms^−1^) between 30 and 90% was measured offline using Clampfit 10.3 (Molecular Devices, Sunnyvale, CA, USA) and normalized to the mean rise/slope of the baseline. Slices were eliminated if an unstable baseline was produced or if the normalized rise/slope dropped >20–50 mV ms^−1^ in an ~10 min period. All the tests were always performed by an experimenter who was unaware of the treatment.

### Primary neuron studies

Cortices from h-tau mouse pups (P0) were isolated and incubated in 1% papain/Hanks balanced salt solution/.5 mmol l^−1^ EDTA without Ca^++^ or Mg^++^(Fisher Scientific, Waltham, MA, USA) as previously described.^14^ Briefly, cells were plated in Neurobasal-A medium (Gibco Life Technologies, Grand Island, NY, USA) plus 10% fetal bovine serum on poly-d-lysine-coated six-well plates at a density of 106 cells per well and kept at 37 °C. At 24 h after plating, medium was removed and replaced with Dulbecco's modified Eagle's medium plus B27 supplements and GlutaMAX (Gibco Life Technologies) to promote neuronal survival and inhibit growth of non-neuronal cells. Neurons were used for experimentation 7 days after plating, when at ~70% confluence and treated with either Neurobasal-A medium with glucose and without glucose for 24 h. After treatment, supernatants were collected, and cells were harvested in lytic buffer for biochemical analyses.

### Data analysis

Unpaired Student's *t*-test (two-sided) was performed using Prism 5.0 (GraphPad Software, La Jolla, CA, USA). All data are presented as mean±s.e.m. Significance was set at *P*<0.05.

## Results

### Effect of glucose deprivation on memory

**Figure 1:**
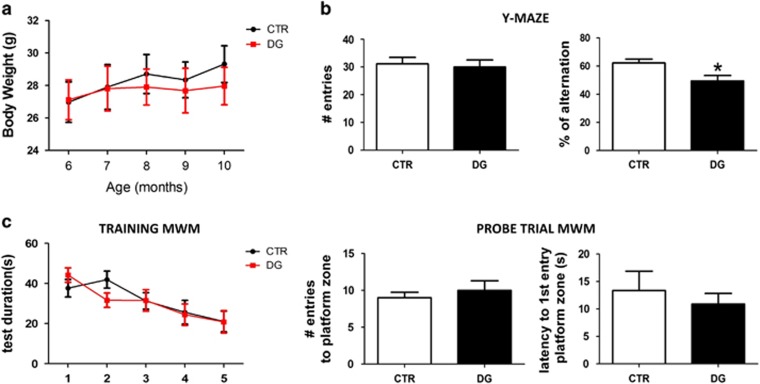
Glucose deprivation impairs cognition in the h-tau mice. (**a**) Body weights (g) of 2-deoxyglucose (DG)-treated and control (CTR) mice were recorded at the beginning of the study and each month until the end when the mice were 10 months old. (**b**) Total number of entries and percentage of alternation in the Y-maze for controls (CTR) and h-tau mice that underwent DG treatment (DG; *P< 0.05). (**c**) Morris water maze test results for controls (CTR) and h-tau mice undergoing DG treatment (DG). Mice were tested initially in the cue test and then trained for 5 days to reach a platform (training phase). After the last training session, for the probe trials phase mice were put back in the water maze and the number of entries to the target platform zone as well as the distance until the first entry in the target platform zone recorded. Results are mean±s.e.m. (*n*=9 mice per group).

Starting at 6 months of age h-tau mice were randomized to receive an intraperitoneal injection of DG or vehicle once a week for 4 months. During the treatment no macroscopic differences between the two groups were observed in terms of general daily and motor activity, food and water intake, social behavior or weight gain (Figure 1a). To assess the effect of glucose deprivation on cognition, at the end of the treatment mice were tested in the Y-maze and Morris water maze. No significant differences were detected among the 2 groups of mice in regard to their general motor activity, as assessed by the number of arm entries in the Y-maze (Figure 1b). However, when we analyzed their exploratory ability the DG group showed a statistically significant lower percentage of alternations compared with the control group (Figure 1b). Animals were then tested in the Morris water-maze paradigm consisting on a visible platform training followed by hidden platform testing with 4 probe trials per day. All mice in each group were able to reach the training criterion within 5 days and no differences were found during the training session as well as in the number of entries and latency to first entry the target platform zone in the probe test trial between the two groups (Figure 1c).

### Glucose deprivation impairs synaptic function

**Figure 2:**
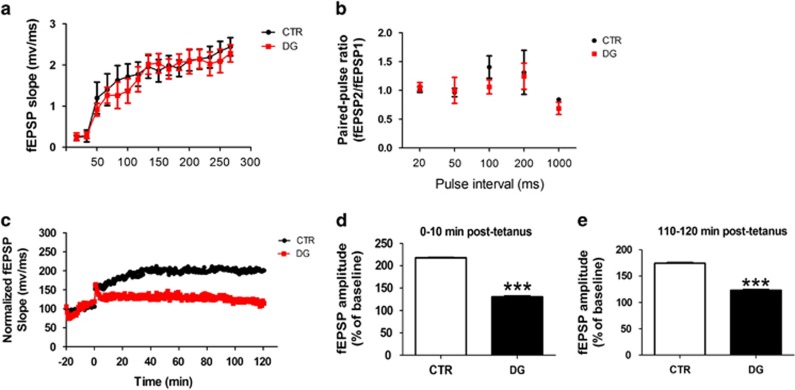
Glucose deprivation impairs synaptic function in the h-tau mice. (**a**) Input/output (I/O) curves and representative field excitatory postsynaptic potentials (fEPSPs) at increasing stimulus strengths (0–300 μA) are shown for CTR and 2-deoxyglucose (DG) treated h-tau mice at 10 months of age. (**b**) Mean fEPSP slopes as a function of interpulse interval between the first and second fEPSPs evoked at CA3–CA1 synapses in slices from the same mice at 20, 50, 100, 200, and 1000 milliseconds in the same animals. (**c**) fEPSP slopes were recorded for 2 h and expressed as the percentage of pretetanus baseline in the same mice. (**d**) Long-term potentiation (LTP) magnitudes expressed as the percentages of baseline for 0 to 10 min post-tetanus (***P<0.001). (**e**) For the same groups of mice, LTP magnitudes expressed as the percentages of baseline for 110 to 120 min post-tetanus (***P<0001). Values represent mean±s.e.m.

Next, we explored the effect of the treatment with DG on synaptic function. To this end, we investigated the basal synaptic transmission in both groups by generating I/O curves and measuring fEPSPs elicited in CA1 by stimulation of the Schaffer collaterals at increasing strength of stimulus intensities. No differences were observed in the I/O curves between the 2 groups of mice (Figure 2a). Measure of short-term plasticity by examining paired-pulse facilitation, which is due to an activity-dependent presynaptic modulation of transmitter release, revealed also no differences between the two groups analyzed (Figure 2b). Finally, we investigated LTP in the CA1 region of the hippocampus, which is thought to be a measure of neuronal plasticity and a major player in cognition. In this test, we found that, compared with CTR, DG-treated mice had a statistically significant reduction in LTP responses (Figure 2c to e).

### Glucose deprivation promotes tau phosphorylation

**Figure 3:**
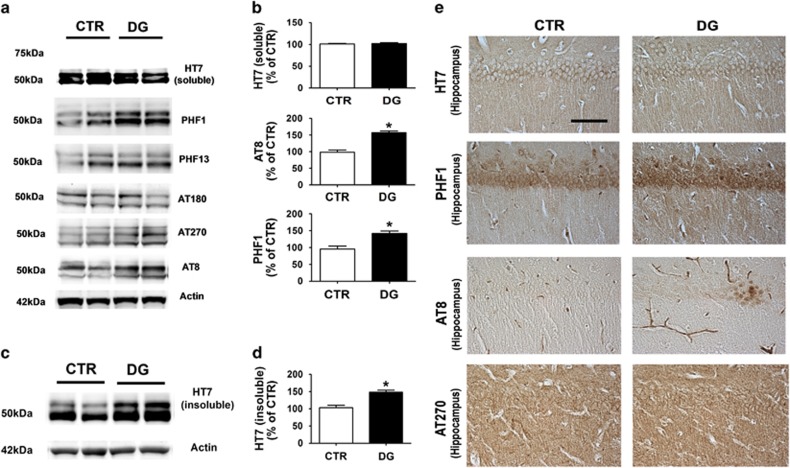
Glucose deprivation increases tau phosphorylation. (**a**) Representative western blot analyses of soluble total tau (HT-7) and phosphorylated tau at residues S396/S404 (PHF-1), S396 (PHF-13), T231/S235 (AT180), T181 (AT270), and S202/T205 (AT8) in brain cortex homogenates of CTR and 2-deoxyglucose (DG)-treated h-tau mice. (**b**) Densitometric analyses of the immunoreactivities shown in the previous panel (*P<0.05). (**c**) Representative western blot analyses of sarkosyl-soluble tau (HT7) in brain cortex homogenates from CTR and DG-treated h-tau. (**d**) Densitometric analyses of the immunoreactivities shown in the previous panel (*P<0.05). (**e**) Representative images of immunohistochemistry analyses for soluble total tau (HT-7) and phosphorylated tau at residues S396/S404 (PHF-1), S202/T205 (AT8), and T181 (AT270) in brain sections of CTR and DG-treated h-tau mice (Scale bar: 100μm). Results are mean±s.e.m.

Brains from the 2 groups of mice were harvested and cortices assayed for the levels of total tau and its phosphorylation at several epitopes. Levels of soluble total tau, as recognized by the antibody HT7, were not different between the treated and untreated mice (Figure 3a). In a similar manner, no differences were noted for tau phosphorylation at Thr181 (AT270), Thr231/ser235 (AT180) and, Ser396 (PHF-13) between the 2 groups (Figure 3a). By contrast, compared with controls DG-treated mice showed an increase in tau phosphorylated at Ser202/Thr205, as recognized by AT8 antibody, and at Ser396/404, as recognized by PHF1 antibody (Figure 3a and b). Immunohistochemical analyses for tau and its phosphorylated isoforms confirmed the biochemical results (Figure 3e). Finally, compared with controls brain homogenates from DG-treated mice displayed a significant increase in the level of total insoluble (formic acid-soluble) tau fraction as recognized by the antibody HT7 (Figure 3c and d).

### Glucose deprivation triggers P38 MAPK kinase activation

**Figure 4:**
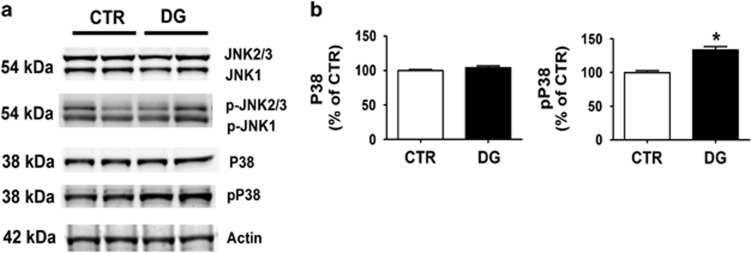
Glucose deprivation triggers P38 MAPK kinase activation. (**a**) Representative western blot analyses of JNK, pJNK, P38 and pP38 in brain cortex homogenates from CTR and 2-deoxyglucose (DG)-treated h-tau mice (*P<0.05). (**b**) Densitometric analyses of the immunoreactivities shown in the previous panel (*P<0.05). Results are mean±s.e.m.

Because we observed changes in tau phosphorylation in the brain cortex region of mice undergoing DG treatment, next we assayed some of the kinases, which have been implicated in post-translational modification of tau secondary to metabolic stress conditions such as JNK and P38 MAPK.^15^ Immunoblot analysis of JNK and its phosphorylated form (pJNK), did not show any significant differences between the 2 experimental groups (Figure 4a). By contrast, although there was no significant difference in the levels of total P38 MAPK between the two groups, compared with controls we observed that brains from DG-treated mice had a significant increase in the levels of its active phosphorylated form pP38 MAPK (Figure 4a and b).

### Glucose deprivation affects synaptic integrity

**Figure 5:**
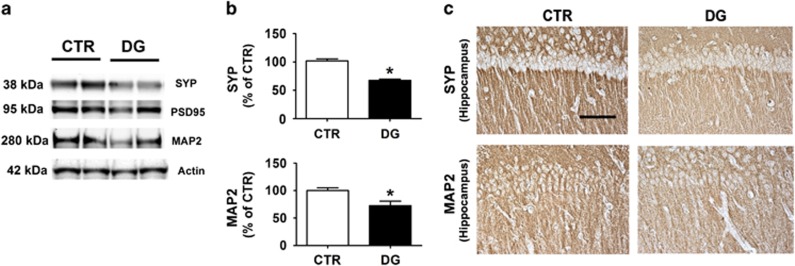
Glucose deprivation induces synaptic pathology in h-tau mice. (**a**) Representative western blot analyses of synaptophysin (SYP), post-synaptic protein-95 (PSD-95), and MAP2 in brain cortex homogenates from CTR and DG-treated h-tau mice (*P<0.05). (**b**) Densitometric analyses of the immunoreactivities shown in the previous panel (*P<0.05; *n*=9 mice per group). Results are mean±s.e.m. (**c**) Representative images of immunohistochemistry analyses for SYP and MAP2 in brain sections of CTR and DG-treated h-tau mice (scale bar: 100μm).

Because changes in tau phosphorylation state and solubility have been correlated with the extent of synaptic loss in AD,^16^ next brain cortices were assayed for steady-state levels of three major synaptic proteins: MAP2, SYP and postsynaptic density protein 95 (PSD-95), indices of pre- and post-synaptic integrity, respectively. As shown in Figure 5a, no differences were observed between the two groups when PSD-95 levels were measured. By contrast, compared with the control group, DG-treated mice displayed a significant decrease in the levels of the main synaptic proteins SYP and the dendritic protein MAP2 (Figure 5a and b). These results were further confirmed in brain sections of the same mice when assessed by immunohistochemical analyses (Figure 5c).

### Glucose deprivation induces apoptosis

**Figure 6:**
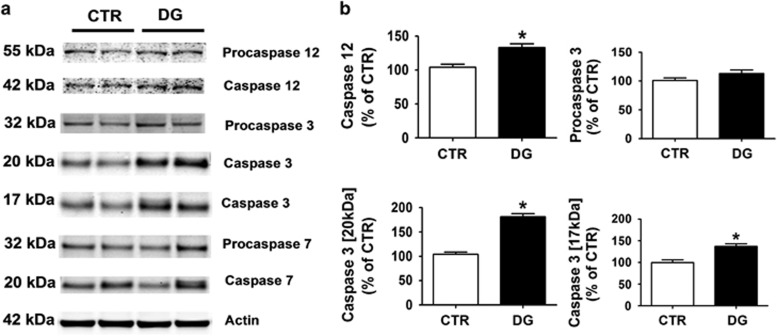
Glucose deprivation-dependent neuronal apoptosis is mediated by caspases 12 and 3. (**a**) Representative western blot analysis for procaspase-12, procaspase-3, procaspase-7, caspase-12, caspase-3, and caspase-7 in brain cortex homogenates from CTR and DG-treated h-tau mice (*P<0.05). (**b**) Densitometric analyses of the immunoreactivities shown in the previous panel (*P<0.05). Results are mean±s.e.m.

Since chronic energy deprivation as well as activation of the P38 MAPK pathway can also lead to cell apoptosis,^17^ we performed immunoblotting analysis for some of the most important caspases, which have been involved in this pathway. As shown in Figure 6, compared with control, DG-treated mice showed a statistically significant increase in the level of the active-cleaved forms of caspase-3 and caspase-12. By contrast, no significant differences were observed between the two conditions when procaspase-12, pro-caspase-7, and caspase 7 steady-state levels were assayed (Figure 6).

### Glucose deprivation modulates tau phosphorylation via P38 MAPK

**Figure 7:**
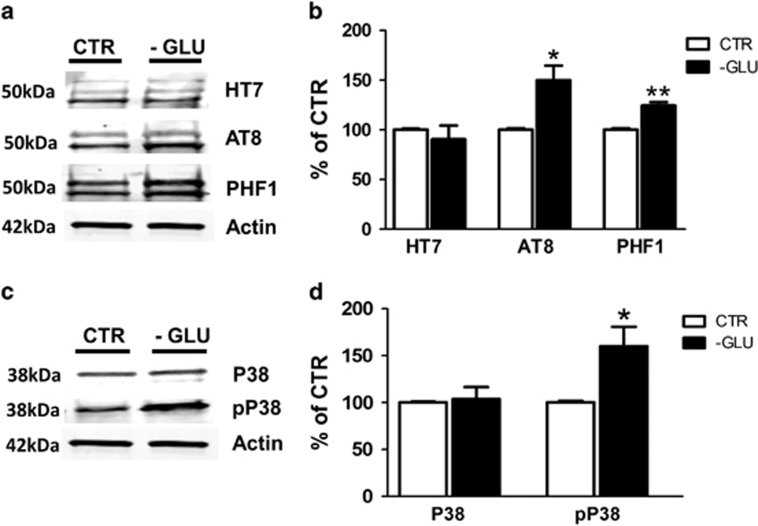
Glucose deprivation modulates tau phosphorylation via P38 MAPK kinase. (**a**) Representative western blots of total tau (HT7) and phosphorylated tau at residues S202/T205 (AT8) and S396/S404 (PHF-1) in primary cortical neuronal cells from h-tau mice incubated with normal medium (CTR) or glucose-deprived (-GLU) medium. (**b**) Densitometric analyses of the immunoreactivities to the antibodies shown in the previous panel (*P<0.05, **P<0.01). (**c**) Representative western blot analyses for P38 and pP38 in primary cortical neurons from h-tau mice incubated with normal medium (CTR) or glucose-deprived (-GLU) medium. (**d**) Densitometric analyses of the immunoreactivities to the antibodies shown in the previous panel (*P<0.05). Values represent mean±s.e.m.

To further confirm our observation, we used primary neuronal cells isolated form the same h-tau transgenic mice. Neurons were incubated overnight in either DMEM with glucose, or DMEM without glucose and then cell lysate collected. Compared with controls, we observed that glucose deprived cells did not show any changes in total tau (HT7) levels (Figure 7). By contrast, we observed that the same cells had a significant increase in tau phosphorylation at epitopes Ser202/Thr205, as recognized by AT8 antibody and, at Ser396/404, as recognized by PHF1 antibody (Figure 7). In addition, similar to the *in vivo* data, we observed a significant increase in the levels of the phosphorylated form of P38 MAPK kinase, but no changes in its total un-phosphorylated form (i.e., P38) (Figure 7).

## Discussion

In recent years, growing experimental evidence has suggested a direct association between altered glucose metabolism, brain function and neurodegeneration.^18, 19^

Consistent data have indeed established a link between systemic metabolic dysfunction, such as diabetes, and dementing disorders, suggesting that their recently observed significant increase in incidence could be in part justified by the worldwide dramatic rise in insulin resistance, obesity and diabetes.^20^ The complexity of this relationship has been more recently underscored by new evidence suggesting that the interaction between altered metabolism and brain dysfunction can be also bidirectional.^21^

In an effort to shed light into such a complicated matter, the implementation of different mouse models has been widely sought. To this end, it is of interest that together with human studies, the investigations using these models have all convened on a common final point: dysregulated glucose levels and impaired energy metabolism secondary to reduced glucose utilization are not only a clinical feature of AD but also important contributors to its pathogenesis by activating the unfolded protein response, increasing Aβ formation and deposition and, in extreme cases, resulting in neuronal death.^22, 23^ Velliquette *et al.*^24^ reported that energy inhibition significantly increased Aβ levels in the Tg2576 mouse model. More recently, this finding was confirmed in an *in vitro* model of energy deficiency and *in vivo*, in two APP transgenic mouse models of AD-like amyloidosis (that is, Tg2576 and 5xFAD mice) in which pharmacological energy deprivation promoted amyloidogenesis via BACE-1-dependent mechanism.^9^ However, since these mice do not develop tau neuropathology it remained to be fully investigated whether energy deprivation was also capable to alter tau metabolism and phosphorylation.

In a recent paper, we investigated the effect of glucose deprivation on tau metabolism using a neuronal cell line, and showed that under this experimental condition cells manifest an increase in tau phosphorylation via the activation of the p38 MAPK.^10^ On the basis of our *in vitro* studies we predicted that the increase in tau phosphorylation in an *in vivo* model of chronic energy depletion would be ultimately responsible for cognitive impairments and synaptic dysfunction.

To test this hypothesis, in the current paper we used a well-established model of impaired energy metabolism, by implementing chronic administration of DG, a synthetic glucose analog in which the C-2 hydroxyl group is substituted by hydrogen. DG competitively inhibits glucose cellular uptake since it uses the same system to enter the cell, the glucose transporters (that is, GLUTs).^25^ Once inside the cell, DG is quickly phosphorylated to form DG-6-phosphate which cannot be further metabolized via the glycolytic pathway and starts accumulating. This event by inhibiting cellular glycolysis at the initial step ultimately results in intra-cellular glucose deprivation.^26^

At the end of the 4 months of treatment, behavioral tests assessing learning and memory were performed in DG-treated mice compared with controls. Interestingly, although we did not observe any significant difference between the two groups in the Morris water maze paradigm, a learning and memory retrieval test, we found that DG-treated mice displayed cognitive impairment as assessed in the Y-maze paradigm, which is considered a reliable measure of spatial working memory in rodents.^27^ Considering that both tests assay hippocampal-dependent memory tasks, we hypothesize that the Y-maze at this particular age is probably a more sensitive test for detecting early behavioral impairments in this transgenic mouse model of tauopathy.^28^

In search for a biochemical substrate of the behavioral data, we evaluated the direct effect of glucose deprivation on synaptic function and transmission by performing electrophysiology studies. First, we observed that between the two groups of mice there were no significant differences in the basal synaptic transmission, indicating that this function is not altered by the treatment. Additionally, no significant differences were noted also when we measured short-term plasticity by examining paired-pulse facilitation, which is secondary to an activity-dependent presynaptic modulation of transmitter release.^29^ This observation suggests that the transient increase in the concentration of intra-terminal calcium produced by an invading action potential is similar between the two groups. However, when we assessed the LTP response, which is a type of neuronal plasticity that is thought to have a major role in learning and memory functions,^30^ we found that DG-treated mice had a statistically significant reduction at 10 min as well as 120 min when compared with the control group.

Considering the age of the mice in our study, we hypothesize that the DG treatment acts as a trigger to exacerbate and accelerate the ongoing pathological changes secondary to the transgene expressed by the mice. Thus, it is known that in the h-tau mice both the behavior deficits as well as LTP changes are typically observed at a later age-time point than the one we have chosen.^11, 28^

Memory deficits were associated with a significant increase in pP38 MAPK-dependent tau phosphorylation at specific epitopes as recognized by the AT8 and PHF1 antibodies along with a significant accumulation of tau in its insoluble form.

Accumulating evidence suggest that P38 MAPK may play an important role in the development of AD pathology.^31, 32^ Several studies have shown that the active form of this kinase is associated with neuritic Aβ-amyloid plaques and tau NFT pathology in postmortem brains of AD patients and that it occurs at very early stage of the disease.^33, 34^ This kinase exists in four different isoforms (α, β, γ, δ) and, when activated by dual phosphorylation at Thr180 and Tyr182, directly promotes tau phosphorylation.^34^ The activation of this kinase can be triggered by many stimuli and stressors such as UV light, heat, osmotic shock, inflammatory cytokines, ER stress and glucose deprivation.^35^ Thus, it is not surprising that under our experimental conditions we observed a specific and selective activation of this kinase. Of interest in the context of our paper, is also the recent report that an increased activity of pP38 MAPK is a significant contributor to LTP inhibition and synaptic dysfunction.^36^

Besides synaptic dysfunction, aberrant accumulation of hyperphosphorylated tau isoforms and its insoluble fibrils can also lead to synaptic loss, typically represented by decreased levels of pre- and post-synaptic protein markers of synaptic integrity.^37^ Thus, confirming this aspect of the neurobiology of phosphorylated tau we found that compared with controls DG-treated h-tau mice had a significant reduction in the steady state levels of 2 major of these synaptic markers, SYP and MAP2, which further explains the deficit in working memory observed in the same animals.

Tau phosphorylation is known to influence not only neuroplasticity, but also neuronal survival by affecting the dendritic/synaptic remodeling seen in the hippocampus as a response to environmental stimuli and stress.^38^ Supporting these observations, in our study we found that compared with controls glucose-deprived mice in addition to higher phosphor-tau displayed biochemical markers of apoptosis, as shown by the increased steady state levels of the cleaved forms of caspase 3 and 12.

Taken together our findings could provide a novel and plausible molecular mechanism linking repeated episodes of hypoglycemia to the development of AD neuropathology.

In fact there are reports that super tight glucose control with insulin Rx increases risk for hypoglycemic episodes, and repeated hypoglycemic episodes have been implicated as part of the diabetes-dementia syndrome.^39^

In summary, the findings presented in this paper provide the first *in vivo* experimental evidence that impaired glucose metabolism and utilization by activating the P38 MAPK kinase pathway triggers tau phosphorylation, neuronal apoptosis, impairs memory, synaptic integrity and function in a relevant transgenic mouse model of tauopathy. Therefore, drugs targeting this kinase in the brain may represent a suitable therapeutic approach for the treatment of both AD and related tauopathies for which impaired glucose utilization is an established risk factor.

## Figures and Tables

**Table 1:** Antibodies used in the study

*Antibody*	*Immunogen*	*Host*	*Application*	*Source*
HT7	aa of human Tau	Mouse	WB/IHC	Pierce
AT8	Peptide containing phospho-S202/T205	Mouse	WB/IHC	Pierce
AT180	Peptide containing phospho-T231/S235	Mouse	WB	Pierce
AT270	Peptide containing phospho-T181	Mouse	WB/IHC	Pierce
PHF-1	Peptide containing phospho-Ser396/S404	Mouse	WB/IHC	Dr Davies P.
PHF-13	Peptide containing phospho-Ser396	Mouse	WB	Pierce
JNK2/3	aa of human JNK2	Rabbit	WB	Cell Signaling
pJNK2/3	Peptide containing phospho-T183/185	Mouse	WB	Cell Signaling
P38	aa of human P38 MAPK	Rabbit	WB	Cell Signaling
pP38	aa around Thr180/182 of human P38 MAPK	Rabbit	WB	Cell Signaling
SYP	aa 221-313 of SYP of human origin	Mouse	WB/IHC	Santa Cruz
PSD95	Purified recombinant rat PSD-95	Rabbit	WB	Cell Signaling
MAP2	Bovine brain microtubule protein	Rabbit	WB/IHC	Millipore
Caspase 12	aa near the N-terminus of caspase 12 of mouse origin	Rabbit	WB	Cell Signaling
Caspase 3	aa 1-227 of procaspase 3 of human origin	Rabbit	WB	Santa Cruz
Caspase 7	aa near the N-terminus of caspase 7 of human origin	Rabbit	WB	Santa Cruz
Actin	aa C-terminus of Actin of human origin	Rabbit	WB	Santa Cruz

Abbreviations: aa, amino acid; IHC, immuno-histochemistry; PSD-95, postsynaptic density protein 95; SYP, synaptophysin; WB, western blot.

## References

[bib1] Iqbal K, Liu F, Gong CX. Tau and neurodegenerative disease: the story so far. Nat Rev Neurol 2016; 12: 15–27. 26635213 10.1038/nrneurol.2015.225

[bib2] Arriagada PV, Growdon JH, Hedley-Whyte ET, Hyman BT. Neurofibrillary tangles but not senile plaques parallel duration and severity of Alzheimer's disease. Neurology 1992; 42: 631–639. 1549228 10.1212/wnl.42.3.631

[bib3] Riley KP, Snowdon DA, Markesbery WR. Alzheimer's neurofibrillary pathology and the spectrum of cognitive function: findings from the Nun Study. Ann Neurol 2002; 51: 567–577. 12112102 10.1002/ana.10161

[bib4] Rissman RA. Stress-induced tau phosphorylation: functional neuroplasticity or neuronal vulnerability? J Alzheimer's Dis 2009; 18: 453–457. 19584431 10.3233/JAD-2009-1153 PMC2906152

[bib5] De Felice FG, Lourenco MV. Brain metabolic stress and neuroinflammation at the basis of cognitive impairment in Alzheimer's disease. Front Aging Neurosci 2015; 7: 94. 26042036 10.3389/fnagi.2015.00094 PMC4436878

[bib6] González-Moreno EI, Cámara-Lemarroy CR, González-González JG, Góngora-Rivera F. Glycemic variability and acute ischemic stroke: the missing link? Transl Stroke Res 2014; 5: 638–646. 25085437 10.1007/s12975-014-0365-7

[bib7] Castellano CA, Nugent S, Paquet N, Tremblay S, Bocti C, Lacombe G et al. Lower brain 18 F-fluorodeoxyglucose uptake but normal 11C-acetoacetate metabolism in mild Alzheimer's disease dementia. J Alzheimer's Dis 2015; 43: 1343–1353. 25147107 10.3233/JAD-141074

[bib8] Lange C, Suppa P, Frings L, Brenner W, Spies L, Buchert R. Optimization of statistical single subject analysis of brain FDG PET for the prognosis of mild cognitive impairment-to-Alzheimer's disease conversion. J Alzheimer's Dis 2015; 49: 945–959. 10.3233/JAD-150814 26577523

[bib9] O'Connor T, Sadleir KR, Maus E, Velliquette RA, Zhao J, Cole SL et al. Phosphorylation of the translation initiation factor eIF2α increases BACE1 levels and promotes amyloidogenesis. Neuron 2008; 60: 988–1009. 19109907 10.1016/j.neuron.2008.10.047 PMC2667382

[bib10] Lauretti E, Pratico' D. Glucose deprivation increases tau phosphorylation via P38 mitogen-activated protein kinase. Aging Cell 2015; 14: 1067–1074. 26219917 10.1111/acel.12381 PMC4693472

[bib11] Andorfer C, Kress Y, Espinoza M, de Silva R, Tucker KL, Duff K et al. Age-dependent impairment of cognitive and synaptic function in the htau mouse model of tau pathology. J Neurosci 2009; 29: 10741–10749. 19710325 10.1523/JNEUROSCI.1065-09.2009 PMC2760256

[bib12] Di Meco A, Lauretti E, Vagnozzi A, Praticò D. Zileuton restores memory impairments and reverses amyloid and tau pathology in aged AD mice. Neurobiol Aging 2014; 35: 2458–2464. 24973121 10.1016/j.neurobiolaging.2014.05.016 PMC4171192

[bib13] Giannopoulos PF, Chu J, Joshi YB, Sperow M, Li JG, Kirby LG et al. 5-lipoxygenase activating protein reduction ameliorates cognitive deficit, synaptic dysfunction, and neuropathology in a mouse model of Alzheimer's disease. Biol Psychiatry 2013; 74: 348–356. 23683389 10.1016/j.biopsych.2013.04.009 PMC3742720

[bib14] Giannopoulos PF, Chu J, Sperow M, Li JG, Yu WH, Kirby LG et al. Pharmacologic inhibition of 5-lipoxygenase improves memory, rescues synaptic dysfunction, and ameliorates tau pathology in a transgenic model of tauopathy. Biol Psychiatry 2015; 78: 693–701. 25802082 10.1016/j.biopsych.2015.01.015 PMC4529386

[bib15] Cheung WD, Hart GW. AMP-activated protein kinase and p38 MAPK activate O-GlcNAcylation of neuronal proteins during glucose deprivation. J Biol Chem 2008; 283: 13009–13020. 18353774 10.1074/jbc.M801222200 PMC2435304

[bib16] Lasagna-Reeves CA, Castillo-Carranza DL, Sengupta U, Clos AL, Jackson GR, Kayed R. Tau oligomers impair memory and induce synaptic and mitochondrial dysfunction in wild-type mice. Mol Neurodegen 2011; 6: 39. 10.1186/1750-1326-6-39 PMC3224595 21645391

[bib17] De la Cadena SG, Hernández-Fonseca K, Camacho-Arroyo I, Massieu L. Glucose deprivation induces reticulum stress by the PERK pathway and caspase-7- and calpain-mediated caspase-12 activation. Apoptosis 2014; 19: 414–427. 24185830 10.1007/s10495-013-0930-7

[bib18] Mergenthaler P, Lindauer U, Dienel GA, Meise A. Sugar for the brain: the role of glucose in physiological and pathological brain function. Trends Neurosci 2013; 36: 587–597. 23968694 10.1016/j.tins.2013.07.001 PMC3900881

[bib19] Sato N, Morishita R. The roles of lipid and glucose metabolism in modulation of β-amyloid, tau, and neurodegeneration in the pathogenesis of Alzheimer disease. Front Aging Neurosci 2015; 7: 199. 26557086 10.3389/fnagi.2015.00199 PMC4615808

[bib20] Stoeckel LE, Arvanitakis Z, Gandy S, Small D, Kahn CR, Pascual-Leone A et al. Complex mechanisms linking neurocognitive dysfunction to insulin resistance and other metabolic dysfunction. F1000Research 2016; 5: 353. 27303627 10.12688/f1000research.8300.1 PMC4897751

[bib21] Biessels GJ, Strachan MW, Visseren FL, Kappelle LJ, Whitmer RA. Dementia and cognitive decline in type 2 diabetes and prediabetic stages: towards targeted interventions. Lancet Diabetes Endocrinol 2014; 2: 246–255. 24622755 10.1016/S2213-8587(13)70088-3

[bib22] Chung J, Yoo K, Kim E, Na DL, Jeong Y. Glucose Metabolic Brain Networks in Early-Onset vs. Late-Onset Alzheimer's Disease. Front Aging Neurosci 2016; 8: 159. 27445800 10.3389/fnagi.2016.00159 PMC4928512

[bib23] Fonseca AC, Ferreiro E, Oliveira CR, Cardoso SM, Pereira CF. Activation of the endoplasmic reticulum stress response by the amyloid-beta 1-40 peptide in brain endothelial cells. Biochim Biophys Acta 2013; 1832: 2191–2203. 23994613 10.1016/j.bbadis.2013.08.007

[bib24] Velliquette RA, O'Connor T, Vassar R. Energy inhibition elevates beta-secretase levels and activity and is potentially amyloidogenic in APP transgenic mice: possible early events in Alzheimer's disease pathogenesis. J Neurosci 2005; 25: 10874–10883. 16306400 10.1523/JNEUROSCI.2350-05.2005 PMC6725876

[bib25] Kurtoglu M, Maher JC, Lampidis TJ. Differential toxic mechanisms of 2-deoxy-D-glucose versus 2-fluoredeoxy-D-glucos in hypoxic and normoxic tumor cells. Antioxid Redox Signal 2007; 9: 1383–1390. 17627467 10.1089/ars.2007.1714

[bib26] Combs DJ, Reuland DS, Martin DB, Zelenock GB, D'Alecy LG. Glycolytic inhibition by 2-Deoxyglucose reduces hyperglycemia-associated mortality and morbidity in the ischemic rat. Stroke 1986; 17: 989–994. 3764973 10.1161/01.str.17.5.989

[bib27] Aggleton JP, Hunt PR. Rawlins JNP. The effects of hippocampal lesions upon spatial and non-spatial tests of working memory. Behav Brain Res 1986; 19: 133–146. 3964405 10.1016/0166-4328(86)90011-2

[bib28] Gerson J, Castillo-Carranza DL, Sengupta U, Bodani R, Prough DS, DeWitt DS et al. Tau oligomers derived from traumatic brain injury cause cognitive impairment and accelerate onset of pathology in htau mice. Neurotrauma 2016; 33: 2034–2043. 10.1089/neu.2015.4262 PMC5116695 26729399

[bib29] Zucker RS, Regehr WG. Short-term synaptic plasticity. Annu Rev Physiol 2002; 64: 355–405. 11826273 10.1146/annurev.physiol.64.092501.114547

[bib30] Kandel ER, Schwartz JH. Molecular biology of learning: modulation of transmitter release. Science 1982; 218: 433–443. 6289442 10.1126/science.6289442

[bib31] Wang Q, Walsh DM, Rowan MJ, Selkoe DJ, Anwyl R. Block of long-term potentiation by naturally secreted and synthetic amyloid beta-peptide in hippocampal slices is mediated via activation of the kinases c-Jun N-terminal kinase, cyclin-dependent kinase 5, and p38 mitogen-activated protein kinase as well as metabotropic glutamate receptor type 5. J Neurosci 2004; 24: 3370–3378. 15056716 10.1523/JNEUROSCI.1633-03.2004 PMC6730034

[bib32] Dai HL, Hu WY, Jiang LH, Li L, Gaung XF, Xiao ZC. p38 MAPK inhibition improves synaptic plasticity and memory in angiotensin II-dependent hypertensive mice. Sci Rep 2016; 6: 27600. 27283322 10.1038/srep27600 PMC4901328

[bib33] Peel AL, Sorscher N, Kim JY, Galvan V, Chen S, Bredesen DE. Tau phosphorylation in Alzheimer's disease: potential involvement of an APP-MAP kinase complex. Neuro Mol Med 2004; 5: 205–218. 10.1385/NMM:5:3:205 15626821

[bib34] Reynolds CH, Betts JC, Blackstock WP, Nebreda AR, Anderton BH. Phosphorylation sites on tau identified by nano-electrospray mass spectrometry: differences *in vitro* between the mitogen activated protein kinases ERK2, c-Jun N-terminal kinase and P38, and glycogen synthase kinase-3beta. J Neurochem 2000; 74: 1587–1595. 10737616 10.1046/j.1471-4159.2000.0741587.x

[bib35] Zarubin T, Han J. Activation and signaling of the p38 MAP kinase pathway. Cell Res 2005; 15: 11–18. 15686620 10.1038/sj.cr.7290257

[bib36] Munoz L, Ammit AJ. Targeting p38 MAPK pathway for the treatment of Alzheimer's disease. Neuropharmacology 2010; 58: 561–568. 19951717 10.1016/j.neuropharm.2009.11.010

[bib37] Jadhav S, Cubinkova V, Zimova I, Brezovakova V, Madari A, Cigankova V et al. Tau-mediated synaptic damage in Alzheimer's disease. Transl Neurosci 2015; 6: 214–226. 28123806 10.1515/tnsci-2015-0023 PMC4936631

[bib38] Kopeikina KJ, Hyman BT, Spires-Jones TL. Soluble forms of tau are toxic in Alzheimer's disease. Transl Neurosci 2012; 3: 223–233. 23029602 10.2478/s13380-012-0032-y PMC3460520

[bib39] Geijselaers SL, Sep SJ, Stehouwer CD, Biessels GJ. Glucose regulation, cognition, and brain MRI in type 2 diabetes: a systematic review. Lancet Diabetes Endocrinol 2015; 3: 75–89. 25163604 10.1016/S2213-8587(14)70148-2

